# Evaluation of the novel liver micronucleus assay using formalin-fixed tissues

**DOI:** 10.1186/s41021-019-0128-5

**Published:** 2019-05-09

**Authors:** Shuichi Hamada, Miyuki Shigano, Satoru Kawakami, Maya Ueda, Hajime Sui, Katsuya Yamada, Soichiro Hagio, Ayaka Momonami, Akihisa Maeda, Yukari Terashima, Wakako Ohyama, Takeshi Morita, Makoto Hayashi

**Affiliations:** 1 Safety Assessment Department, Nonclinical Research Center, Drug Development Service Segment, LSI Medience Corporation, 14-1, Sunayama, Kamisu-shi, Ibaraki, 314-0255 Japan; 2 Asahi Kasei Pharma Corporation, 632-1 Mifuku, Izunokuni-shi, Shizuoka, 410-2321 Japan; 3 BioSafety Research Center Inc., 582-2 Shioshinden, Iwata-shi, Shizuoka, 437-1213 Japan; 4 Food and Drug Safety Center, 729-5 Ochiai, Hadano-shi, Kanagawa 257-8523 Japan; 5 Mitsubishi Tanabe Pharma Corporation, 2-2-50 Kawagishi, Toda-shi, Saitama, 335-8505 Japan; 6 Nissan Chemical Corporation, 1470 Shiraoka, Shiraoka-shi, Saitama, 349-0294 Japan; 7 Suntory MONOZUKURI Expert Ltd., 8-1-1 Seikadai, Seika-cho, Soraku-gun, Kyoto, 619-0284 Japan; 8 Toray Industries Inc., 6-10-1 Tebiro, Kamakura-shi, Kanagawa 248-8555 Japan; 9 Kissei Pharmaceutical Co., Ltd., 2320-1 Maki, Hotaka, Azumino-shi, Nagano, 399-8305 Japan; 10 Yakult Honsha Co., Ltd., 5-11 Izumi, Kunitachi-shi, Tokyo, 186-8650 Japan; 11 National Institute of Health Sciences, 3-25-26 Tonomachi, Kawasaki-shi, Kanagawa 210-9501 Japan; 12 makoto international consulting, 4-23-3-1 Kamiimaizumi, Ebina-shi, Kanagawa 243-0431 Japan

**Keywords:** Micronucleus assay, Liver, Formalin-fixed tissue, Collagenase, Hepatocyte

## Abstract

**Background:**

The repeated-dose liver micronucleus (RDLMN) assay is an effective and important in vivo test for detecting genotoxic compounds, particularly for those that require metabolic activation to show genotoxicity. In a collaborative study by the Collaborative Study Group for the Micronucleus Test (CSGMT)/The Japanese Environmental Mutagen Society (JEMS) – Mammalian Mutagenicity Study Group (MMS), micronucleus induction of 22 chemicals with the RDLMN assay employing the collagenase digestion method was examined and reported on. Recently, we have developed a method which enables retrospective evaluation of micronucleus induction in formalin-fixed liver tissues (the formalin-fixed method) obtained in general toxicity studies completed in the past. Using this method, we were able to easily evaluate clastogenic potential of chemicals from the formalin-fixed tissues obtained in the general toxicity studies.

In this study, to evaluate the usefulness of the formalin-fixed method, we have conducted a liver micronucleus assay using the formalin-fixed liver samples obtained from the above collaborative study (18 of 22 test chemicals) and carried out a comparison with the results obtained by the collagenase digestion method.

**Results:**

Comparison of the collagenase digestion and formalin-fixed methods was conducted using the results of the micronucleus assays with a total of 18 test chemicals which included 12 genotoxic hepatocarcinogens (Group A), 4 genotoxic carcinogens but not liver targeted (Group B), and 2 nongenotoxic hepatocarcinogens (Group C). The formalin-fixed method obtained the similar results as the collagenase digestion method in 10 out of the 12 chemicals of Group A, and all chemicals of Group B and Group C. Although the results were statistically contradictive due to different levels of concurrent negative control, the 2 other chemicals of Group A showed comparable responses between the two methods.

**Conclusion:**

The present study shows that the formalin-fixed method is capable of detecting liver carcinogens with sensitivity equal to or higher than that of the collagenase digestion method. We recommend use of the formalin-fixed method because of its capability of enabling retrospective evaluation of micronucleus induction in the formalin-fixed liver tissues obtained in general toxicity studies completed in the past.

## Introduction

Although the liver is not targeted in the routine micronucleus assay, the liver is an important tissue in general toxicology studies and also in carcinogenicity bioassays because test chemicals are metabolized and on occasion activated with toxicological significance in the liver. It is reported that genotoxic rodent hepatocarcinogens [1] that require metabolic activation [2, 3] and/or are not detectable in rodent routine erythrocyte micronucleus assays [4, 5] are detectable by the liver micronucleus assay.

The micronucleus assay using the liver, which is the main organ for drug metabolism, has been known to be important but not widely used because hepatocyte (HEP) proliferation in adult rats is slow and thus micronuclei are difficult to produce. To overcome this shortcoming, partial hepatectomy [6–8], mitogen treatment [9, 10], and the use of juvenile rats [11–14] have been introduced to the assays. All these methods have disadvantages which include complex surgical procedures, decreased metabolic activity due to the partial hepatectomy [15], risk of drug interactions for mitogen treatment [16], and a lack of maturation for metabolic activation in juvenile animals [17]. Recently, we have developed a new method, the repeated-dose liver micronucleus (RDLMN) assay, to evaluate liver micronucleus through repeated administration of test chemicals, e.g., 14-day or 28-day repeated-dose treatments [18]. This method is expected to produce an accumulation of micronucleated hepatocytes (MNHEPs) through long-term continuous treatment, although HEP turnover is slow [18].

The advantages of liver micronucleus assay are made more obvious when it is incorporated into general toxicity studies. In a recently improved formalin-fixed method, procedures to prepare samples for liver micronucleus assays have been provided [19]. Because this method enables retrospective evaluation of micronucleus induction in the formalin fixed liver tissues obtained in general toxicity studies completed in the past, clastogenic potential of chemicals from the materials obtained in the general toxicity studies are able to be easily evaluated.

In a collaborative study by CSGMT/JEMS MMS, micronucleus induction of 22 test chemicals with the RDLMN assay using the collagenase digestion method [1] was examined and reported on. In this study, the micronucleus induction of 18 of those 22 test chemicals has been reexamined using the formalin-fixed liver samples, and the results have been compared with the former collaborative study which employed the collagenase digestion method.

## Materials and methods

### Formalin-fixed liver tissues

Five-year-old formalin (10% phosphate-buffered) fixed liver tissues of a previous collaborative study by CSGMT/JEMS MMS were used to evaluate the micronucleus induction of 18 chemicals with the RDLMN assay by the collagenase digestion method [20–36]. The 18 test chemicals consisted of 12 genotoxic hepatocarcinogens (Group A), 4 genotoxic carcinogens but not liver targeted (Group B), and 2 nongenotoxic hepatocarcinogens (Group C) (Table 1).

**Table 1:** Chemical profiles used in the collaborative study by CSGMT/JEMS MMS

Group	Chemical	Abbreviation	CAS no.	Chemical class
Group A	Dimethylnitrosoamine	DMN	62–75-9	nitroso compound
	*N*-Nitrosopyrrolidine	NPYR	930–55-2	nitroso compound
	*N*-Nitrosodipropylamine	NDPA	621–64-7	nitroso compound
	2,4-Dinitrotoluene	2,4-DNT	121–14-2	aromatic nitro compound
	Quinoline	QUN	91–22-5	heterocyclic compound
	*p*-Dimethylaminoazobenzene	DAB	60–11-7	azo compound
	2-Nitropropane	2-NP	79–46-9	alkyl nitro compound
	Monocrotaline	MCT	315–22-0	alkaloid
	*N*-Nitrosomorpholine	NMOR	59–89-2	nitroso compound
	2-Acetylaminofluorene	2-AAF	53–96-3	aromatic amine
	Sudan I (C.I.solvent yellow 14)	Sudan I	842–07-9	azo compound
	Thioacetamide	TAA	62–55-5	thioamide
Group B	Cyclophosphamide H_2_O	CP	6055-19-2	bis compound
	Potassium bromate	KBrO_3_	7758-01-2	inorganic metal compound
	*N*-Methlyl-*N′*-nitro-*N*-nitrosoguanidine	MNNG	70–25-7	nitroso compound
	Methyl methanesulfonate	MMS	66–27-3	alkyl sulfonate
Group C	Clofibrate	CFB	637–07-0	chlorophenoxy compound
	Methapyrilene HCl	MP	135–23-9	ethylene diamine

Group A: Genotoxic hepatocarcinogens, Group B: genotoxic carcinogens but not liver targeted, Group C: nongenotoxic hepatocarcinogens

In the previous collaborative study [20–36], male Crl:CD(SD) rats purchased from Charles River Japan Inc. (Atsugi, Hino or Tsukuba, Japan) were 6 weeks old at the beginning of dosing. They were housed in an air-conditioned room with a 12-h light/dark cycle and given free access to food and drinking water. The animal experiments were approved by the Institutional Animal Care and Use Committee of each testing facility prior to conducting the experiments. The rats (5/group) were administered each chemical by oral gavage in a repeated dosing regimen for 14 or 28 consecutive days. Twenty-four hours after the last administration for each time point, rats were euthanized under thiopental anesthesia. The livers were then removed from the rats and a part of each liver was used for the liver micronucleus assay employing the collagenase digestion method as previously reported [20–36]. Residual tissues were immersed into 10% phosphate-buffered formalin, and stored for approximately 5 years. They were then provided for the present investigation.

### Preparation of hepatocyte suspensions

HEP-specimens were prepared from the formalin-fixed liver tissues with a slightly modified version of the previously reported method [16, 19]. In brief, a small portion of the fixed-liver tissue was cut into approximately 3 mm-cubes with a razor and thoroughly washed with water. Approximately ten cubes were incubated in approximately 15 mL of 12 M aqueous solution of potassium hydroxide (KOH; Wako Pure Chemical Industries, Ltd., Osaka, Japan) at room temperature for 16 h and then washed thoroughly with water to remove the KOH-solution. The tissue cubes were then mashed, filtered through a cell strainer (pore size: 100 μm), and suspended with water to disperse HEPs. The HEP-suspensions were centrifuged at 50×*g* for 5 min and washed with 10% phosphate-buffered formalin. Centrifugation and washing steps were repeated 3 times or more. The pellet of the HEPs was suspended with 10% phosphate-buffered formalin to prepare an HEP-suspension.

### Fluorescent dyes and reagents

Fluorescent dye, SYBR® Gold (SYGO; 10,000× concentrate in dimethyl sulfoxide) purchased from Life Technologies, Inc. (Carlsbad, CA, USA), was used to stain the isolated HEPs from the formalin-fixed tissue. One mol/L (M) of Tris-hydrochloride (Tris-HCl; pH 7.5) and 0.5 M of ethylenediamine tetraacetic acid (EDTA; pH 8.0) purchased from Wako Pure Chemical Industries, Ltd. (Osaka, Japan) were mixed to prepare a TE buffer (10 mM Tris-HCl and 1 mM EDTA, pH 7.5–8.0). SYGO was diluted 2-fold with the TE buffer. Just before microscopic observation, the prepared HEP-suspension was mixed and stained with the same volume of a solution containing SYGO at half the original concentration. The mixtures were dropped onto clean glass slides and spread with coverslips.

### Microscopic observation and statistical analysis

Each of the slide specimens stained with SYGO was observed under a fluorescent microscope with B-excitation filter (wavelength: 420-490 nm). Two thousand parenchymal HEPs were analyzed, and the number of MNHEPs was recorded [1, 18]. At the same time, the number of mitotic phase cells among the 2000 HEPs was also recorded to calculate the mitotic index (MI).

Differences in the incidence of MNHEPs between groups of test chemicals and vehicle controls were analyzed by the conditional binomial test reported by Kastenbaum and Bowman [37] at significance levels of 5 and 1%. The proportions of MI between the treated and control groups were analyzed using Dunnett’s test. Positive/Negative status was determined based mainly on the statistical analysis of MNHEP incidence to assess the biological relevance of MNHEPs, i.e., the historical control at the laboratory where the study was conducted, as well as the dose-response relationship. The judgment of biological relevance was made in a meeting of the organizing committee of this project.

The data obtained from the formalin-fixed method were compared to the data from the collagenase digestion method in the collaborative study by CSGMT/JEMS MMS [1].

## Results

### Group A chemicals (genotoxic hepatocarcinogens)

**Fig. 1:**
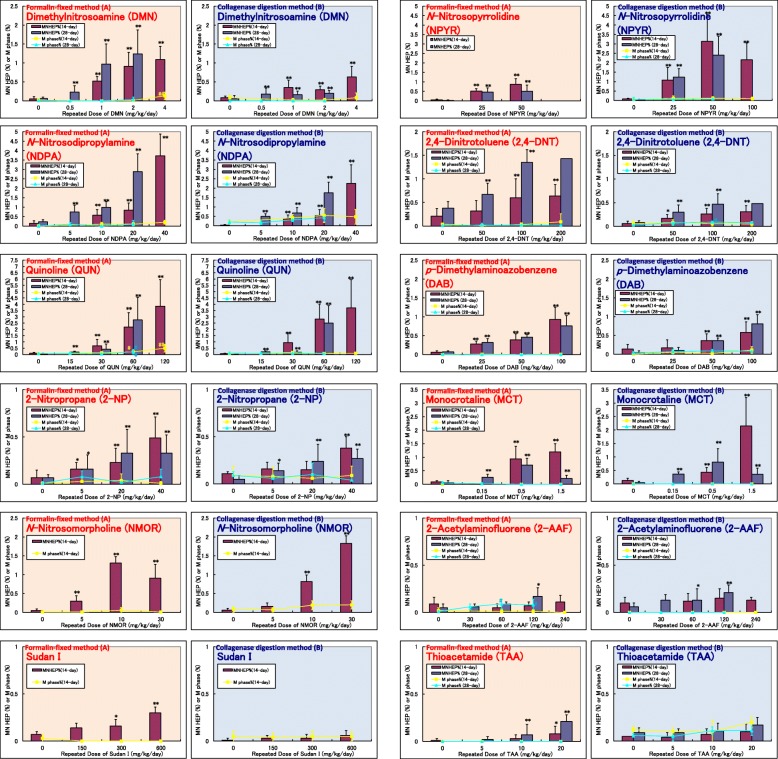
Comparison of RDLMN assay results of formalin-fixed method and collagenase digestion method using Group A chemicals. Incidences of MNHEPs (%); Comparison between formalin-fixed method (**A**) and collagenase digestion method reported by Hamada et al. [1] (**B**) in rats treated with Group A chemicals for 14 or 28 days. As for the 28-day RDLMN assay of NDPA using collagenase digestion method, it was conducted by Kissei Pharmaceutical Co., Ltd. as a collaborative study by CSGMT/JEMS MMS immediately after publication of a report by Hamada et al. [1]. Values are presented as the mean and SD. Differences in the incidences of MNHEPs between the test and vehicle control groups were analyzed by the Kastenbaum and Bowman test at significance levels of 5 and 1% (*: *P* < 0.05, **: *P* < 0.01). Differences in the incidences of mitotic phase cells between the test and vehicle control groups were analyzed by Dunnett’s multiple comparison test at significance levels of 5 and 1% (#: *P* < 0.05, ##: *P* < 0.01). Group A: genotoxic hepatocarcinogen

We reevaluated 12 chemicals of Group A using the formalin-fixed method. As a result, all 12 chemicals were found to be positive for liver micronucleus induction (Fig. 1). When compared to the collagenase digestion method employed in the previous collaborative study, the formalin-fixed method induced almost the same levels of micronuclei in most of the chemicals as the collagenase digestion method.

Relatively higher frequencies of MNHEPs were observed in DMN, NDPA, and 2,4-DNT in the formalin-fixed method, while the same trend was observed in NPYR in the collagenase digestion method. Different results were obtained in TAA and Sudan I, i.e., positive in the formalin-fixed method but negative in the collagenase digestion method.

As for MI, MI evaluated in this study by the formalin-fixed method was 0 to 0.08% in the negative control group and 0 to 0.63% in the test chemical treated group, which was equivalent to that determined previously by the collagenase digestion method (0 to 0.12% in the negative control group, 0 to 0.55% in the test chemical treated group) [1].

### Group B (genotoxic carcinogens but not liver targeted) and Group C chemicals (nongenotoxic hepatocarcinogens)

**Fig. 2:**
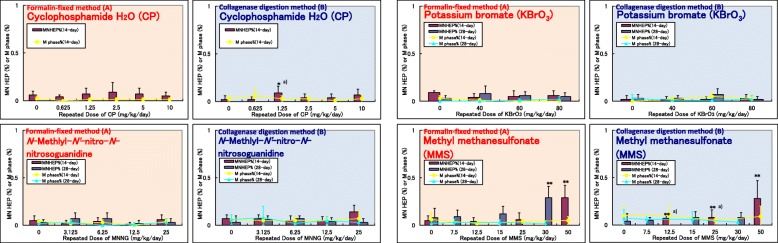
Comparison of RDLMN assay results of formalin-fixed method and collagenase digestion method using Group B chemicals. Incidences of MNHEPs (%); Comparison between formalin-fixed method (**A**) and collagenase digestion method reported by Hamada et al. [1] (**B**) in rats treated with Group B chemicals for 14 or 28 days. Values are presented as the mean and SD. Differences in the incidences of MNHEPs between the test and vehicle control groups were analyzed by the Kastenbaum and Bowman test at significance levels of 5 and 1% (*: *P* < 0.05, **: *P* < 0.01). Differences in the incidences of mitotic phase cells between the test and vehicle control groups were analyzed by Dunnett’s multiple comparison test at significance levels of 5 and 1% (#: *P* < 0.05, ##: *P* < 0.01). a): Statistically significant but judged as negative because the values were within the range of the background data of negative controls in the laboratory where the MN observation was conducted. Group B: genotoxic carcinogens but not liver targeted

**Fig. 3:**
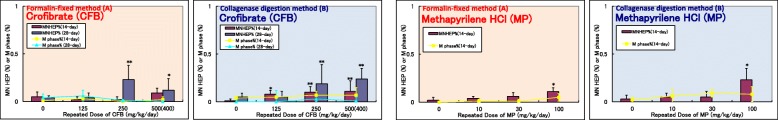
Comparison of RDLMN assay results of formalin-fixed method and collagenase digestion method using Group C chemicals. Incidences of MNHEPs (%); Comparison between formalin-fixed method (**A**) and collagenase digestion method reported by Hamada et al. [1] (**B**) in rats treated with Group C chemicals for 14 or 28 days. Values are presented as the mean and SD. Differences in the incidences of MNHEPs between the test and vehicle control groups were analyzed by the Kastenbaum and Bowman test at significance levels of 5 and 1% (*: *P* < 0.05, **: *P* < 0.01). Differences in the incidences of mitotic phase cells between the test and vehicle control groups were analyzed by Dunnett’s multiple comparison test at significance levels of 5 and 1% (#: *P* < 0.05, ##: *P* < 0.01). Group C: nongenotoxic hepatocarcinogens

In Group B (4 chemicals) and Group C (2 chemicals), the formalin-fixed method showed almost the same levels of micronucleus induction in all chemicals as the collagenase digestion method (Figs. 2 and 3).

As for MI, MI evaluated in this study by the formalin-fixed method was 0 to 0.06% in the negative control group and 0 to 0.06% in the test chemical treated group, which was equivalent to that determined previously by the collagenase digestion method (0 to 0.07% in the negative control group, 0 to 0.09% in the test chemical treated group) [1].

### Performance of the RDLMN assay

**Fig. 4:**
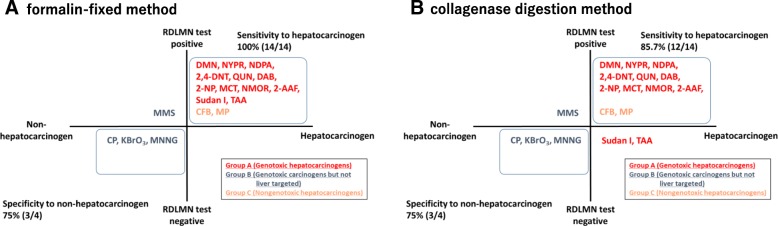
Performance of the RDLMN assay: Comparison between formalin-fixed method (**A**) versus collagenase digestion method (**B**). The data of collagenase digestion method were reported by Hamada et al. [1]. Sensitivity to hepatocarcinogen (%) = (the number of chemicals that showed positive results in RDLMN assay / the number of hepatocarcinogens tested)× 100. Specificity to non-hepatocarcinogen (%) = (the number of chemicals that showed negative results in RDLMN assay / the number of non-hepatocarcinogens tested)× 100

The performance of the RDLMN assay is shown in Fig. 4. The sensitivity to hepatocarcinogens was determined to be 85.7% (12/14) by the collagenase digestion method and 100% (14/14) by the formalin-fixed method. Moreover, the specificity to hepatocarcinogens was 75% (3/4) in both methods.

## Discussion

As predicted, similar results were obtained in the collagenase digestion and formalin-fixed methods in 10 out of 12 chemicals of Group A, all four chemicals of Group B, and both chemicals of Group C.

Sudan I and TAA showed negative in the collagenase digestion method while positive in the formalin-fixed method. Sudan I has been reported negative in in vitro chromosome aberration tests [38], positive in short-term bone marrow micronucleus assays [39], and positive for liver carcinogenicity [40]. In the previous study [1, 29], Sudan I showed a tendency, though slight, toward a dose-dependent increase in liver micronucleus induction observed by the collagenase digestion method, though found negative statistically. Histopathological examination showed a remarkable HEP hypertrophy [1, 29], which indicated a possibility that collagenase treatment under such conditions may injure HEPs, leading to low sensitivity (i.e., negative result) in liver micronucleus assays. For evaluation of chemicals with strong hepatotoxicity, the formalin-fixed method which forms a single cell after formalin fixation and has lower possibility to injure HEPs as compared to the collagenase digestion method is considered more appropriate. As for TAA, the levels of micronucleus induction were comparable in the treatment groups between the two methods, but lower in the negative control of formalin-fixed samples than that of collagenase digestion ones, suggesting that the difference was due to the effect of negative control. In most of the chemicals, micronucleus induction determined by the formalin-fixed method was equivalent to or relatively higher than that determined by the collagenase digestion method except for NPYR, for which collagenase digestion method showed a higher micronucleus induction than the formalin-fixed method. In some chemicals, same results were obtained as to the positive and negative for micronucleus induction; however, the micronucleus induction (%) varied largely by more than 2 times between the collagenase digestion method and the formalin-fixed method. These are possibly due to the testing facilities and observers being different for each method and difference in the part of the liver where samples were collected; however, the exact cause remains unclear and further investigation is considered necessary.

As a result, sensitivity to hepatocarcinogens was slightly higher (100% [14/14]) in the formalin-fixed method as compared to the collagenase digestion method (85.7% [12/14]); however, the specificity to non-hepatocarcinogens did not differ between the two methods. This suggests that the formalin-fixed method has the capability of enabling detection of liver carcinogens equal to or higher than the collagenase digestion method.

MI used as an indicator of cytotoxicity was extremely low in the collagenase digestion method and formalin-fixed method, which suggested that MI is not appropriate as an indicator of cytotoxicity in the RDLMN assay. The cause of the low MI is possibly due to difference in the principals of evaluation, in which accumulation of micronucleus inducers is evaluated over the period of repeated dosing for micronucleus induction, while the number of mitotic cells over several hours before necropsy is evaluated for MI.

An integrated study that can evaluate multiple toxicity indices in the same individual animal is the ideal form of toxicity study. The formalin-fixed method has made it dramatically easier to conduct RDLMN assays by using the liver collected from the animals used in the general toxicity studies. Furthermore, it is possible to conduct a retrospective evaluation of micronucleus induction using formalin-fixed specimens of past toxicity studies. In this context, histopathological examination that is usually conducted in general toxicity studies would give direct information about cytotoxicity and HEP proliferation and indirect information about chemical exposure (more directly with toxicokinetic analysis in the case of pharmaceuticals).

Currently, evaluation by the formalin-fixed method has been commenced not only with the liver but also with the digestive tract, which is considered an effective method to facilitate sharing experimental animals between general toxicity and genotoxicity studies.

## Conclusion

The present study shows that the formalin-fixed method has the capability to enable detection of micronucleus induction in HEPs equal to or higher than the collagenase digestion method. We recommend use of the formalin-fixed method not only for the above reason but also for the fact that it allows retrospective evaluation of micronucleus induction in formalin fixed liver tissues obtained in general toxicity studies completed in the past.

## Abbreviations

2,4-DNT: 2,4-dinitrotoluene
2-AAF: 2-acetylaminofluorene
2-NP: 2-nitropropane
CFB: clofibrate
CP: cyclophosphamide H_2_O
CSGMT: the Collaborative Study Group for the Micronucleus Test
DAB: *p*-dimethylaminoazobenzene
DMN: dimethylnitrosoamine
EDTA: ethylenediamine tetraacetic acid
HEP: hepatocyte
JEMS: The Japanese Environmental Mutagen Society
KBrO_3_: potassium bromate
MCT: monocrotaline
MI: mitotic index
MMS: Mammalian Mutagenicity Study Group
MMS: methyl methanesulfonate
MNHEP: micronucleated hepatocyte
MNNG: *N*-methlyl-*N′*-nitro-*N*-nitrosoguanidine
MP: methapyrilene HCl
NDPA: *N*-nitrosodipropylamine
NMOR: *N*-nitrosomorpholine
NPYR: *N*-nitrosopyrrolidine
QUN: quinoline
RDLMN assay: repeated-dose liver micronucleus assay
SYGO: SYBR® Gold
TAA: thioacetamide
TE: 10 mM Tris-HCl and 1 mM EDTA
Tris-HCl: Tris-hydrochloride
